# The Risk-Taking and Self-Harm Inventory for Adolescents: Validation of the Italian Version (RTSHIA-I)

**DOI:** 10.3390/bs13040321

**Published:** 2023-04-09

**Authors:** Annalisa Valle, Giulia Cavalli, Laura Miraglia, Edoardo Alfredo Bracaglia, Peter Fonagy, Cinzia Di Dio, Antonella Marchetti

**Affiliations:** 1 Research Unit on Theory of Mind, Department of Psychology, Università Cattolica del Sacro Cuore, 20123 Milano, Italy; 2 Research Department of Clinical, Educational and Health Psychology, University College, UCL, London WC1E 6BT, UK

**Keywords:** risk-taking, self-harm, assessment, emotion regulation, internalizing/externalizing traits

## Abstract

The aim of the present paper is to establish the factorial validity and reliability of the Risk-Taking and Self-Harm Inventory for Adolescents (RTSHIA), proposed by Vrouva and colleagues in 2010, in an Italian sample. The RTSHIA measures both Risk-Taking and Self-Harm behavior in adolescents. We administered the scale to a total of 1292 Italian adolescents from 9th to 12th grade; to verify the validity of the scale, we also assessed emotion regulation and psychopathological traits. The exploratory factor analysis (EFA) (N = 638) and the confirmatory factor analysis (CFA) (N = 660) confirmed the original two-factor structure of the RTSHIA (Risk-Taking and Self-Harm). The only differences in the Italian version of the RTSHIA (RTSHIA-I) were that one item was moved from the original Risk-Taking factor to the Italian Self-Harm factor, and another item that was not included in the original RTSHIA is now part of the Risk-Taking factor in the Italian version. The reliability of the RTSHIA-I is also confirmed, and both factors correlate with emotion regulation and externalizing/internalizing traits. Our results suggest that the RTSHIA-I is a useful tool for assessing Risk-Taking and Self-Harm behaviors in Italian adolescents, and the correlational patterns indicate that these behaviors may be related to difficulties in mentalization skills.

## 1. Introduction

Risk-Taking (RT) and Self-Harm (SH) are phenomena that can occur throughout the lifespan but typically appear during adolescence and can have long-lasting effects [1]. Risk-taking is defined as voluntary participation in any behavior that carries some probability of undesirable results [2]. It refers to a range of behaviors that can compromise the health and well-being of adolescents directly or indirectly, with repercussions that can affect the rest of their lives. Risk-taking behavior includes risky driving [3], promiscuous and unsafe sexual practices (for a review, see [4]), alcohol and drug abuse [5], and criminal conducts [2,6]. It has been suggested that self-regulatory competence, i.e., the ability to control, modify, and adapt one’s emotions, impulses, or desires [7], may influence the degree of one’s risk-taking propensity. Indeed, poor self-regulatory control would increase the likelihood of risk participation [8]. Additionally, impulsiveness driven by emotions, which is closely related to poor emotion regulation, is considered a risk factor for RT behaviors, along with a low level of psychosocial well-being, exposure to stressful events [9], and sensation seeking [10]. In contrast, cognitive regulation plays a role in RT as it is common to observe failures of executive function skills, such as inhibition, planning, and maintaining attention when youth engage in reckless behaviors [11]. Lauriola and colleagues [12] also assume that affective risky decisions—decisions in an affective context where emotions are at play—are influenced by a cognitive limitation in understanding and labeling emotions, as well as maladaptive psychological functioning.

Self-harm is defined as the deliberate infliction of direct physical harm without any conscious suicidal intent [13,14]. Self-harm covers a broad spectrum of behaviors ranging from different types of non-suicidal self-injury [15,16], such as cutting, burning, carving, severe scratching, and interference with wound healing. This phenomenon typically starts in early adolescence, around 12–14 years of age, and increases in late adolescence and young adulthood. However, the prevalence of SH is not uniform due to the use of different definitions and research methods. Two independent systematic reviews reported a lifetime prevalence of 17.2% and 18% in adolescents, respectively [17,18], comparable to the 16.9% identified by Gillies and colleagues [19] and 13.3% reported by Evans and colleagues [20]. With respect to the Italian situation, Cerutti and colleagues [21] found that 42% of Italian adolescents experience SH behavior. This phenomenon appears in both clinical and non-clinical populations. Uh and colleagues [22] found that both groups are characterized by low self-esteem, and the non-clinical group showed risk factors such as the tendency to RT, a lack of social support, and peer-related problems. Other risk factors are emotion regulation problems, exposure to potentially traumatic events, and perceived isolation from family and society [23,24]. Cerutti and colleagues [21] assume that for a non-clinical population, the basis of the SH behavior could be an emotional difficulty, specifically the inability to express, externalize, and share one’s emotions. Attempts to manage stressful internal states using poor emotion regulation ability result in both inward- and outward-directed behavior [25], underlying internalizing traits (depression and anxiety) and externalizing traits (such as dissociation and stressful life events) [21] (for a review, see also [26]). Similarly, a lack of mentalization—the capacity to understand that behaviors are underlain by feelings and thoughts—is considered one of the causes of SH behavior [27]. In this complex framework, SH assumes a coping function that adolescents use when faced with emotionally unbearable situations [28] or when they feel unable to express their distress in different ways. Negative life events, such as changes in family, work, and/or study, can also impact the frequency of SH behavior, causing negative psychological and physiological outcomes [29]. One example is the COVID-19 pandemic, which has affected the psychosocial environment of vulnerable children and adolescents by causing loss of lives, isolation, contact restrictions, and disruption of daily rhythms, particularly eating and sleeping routines. In this regard, Mucci and colleagues [30] and Beghi and colleagues [31] found that non-suicidal self-injury significantly increased among Italians during the lockdown periods in 2020 and 2021.

Vrouva and colleagues [32] suggest that RH and SH should be studied together due to their important similarities. Firstly, both originate during adolescence when youths desire to experience something subjectively perceived as desirable, regardless of consequences. This critical period is characterized by vulnerability, weak cognitive control, high emotional and stress reactivity, poor decision-making ability, and consequence appraisal [2]. Moreover, both RT and SH involve the body as a central role player since it is the immediately available vehicle for one’s emotional, expressive, and communicative needs [33]. Risk-taking and self-harm behaviors also share a regulatory function: SH behavior can be functional in regulating and coping with emotions, and similarly, RT propensity may be influenced by emotional regulatory competencies and becomes a coping strategy for adolescents who cannot manage intense emotions autonomously [8,11].

Despite their similarities, significant differences exist between RT and SH [32]. Firstly, in SH, physical pain and injuries are direct and intentional, while in RT, physical harm may be the consequence of the behavior, but not the conscious aim. Moreover, SH usually takes place in solitude, whereas RT behaviors are subject to peer influence [34]. Finally, RT has been considered an adaptive behavior that is normative, biologically driven, and linked to developmental tasks such as autonomy and exploration [35]. On the contrary, SH is a dysfunctional strategy that adolescents adopt to control their intense negative emotions and the difficult situations they are living in [28]. Consequently, whereas RT is linked to a variety of moods, including euphoria, SH is primarily driven by emotional distress.

Due to the potential impact that these behaviors can have on a person’s life, it is appropriate for researchers and clinicians to have empirically validated measures that can detect these phenomena during adolescence [17]. For this purpose, Vrouva and colleagues [32] designed a self-report questionnaire, the *Risk Taking and Self-Harm Inventory for Adolescents (RTSHIA)*. The questionnaire comprises 26 items divided into 2 factors, RT and SH. The RT factor consists of eight items and refers to putting oneself in dangerous and transgressive situations, adopting behaviors of substance dependence (alcohol, smoking, and drugs), and engaging in dangerous sexual conduct. The SH factor consists of 18 items and investigates the presence of various self-harming behaviors, including cuts, burns, bites, bruises, tearing of one’s hair, and drug abuse. Respondents are required to indicate the frequency of the behaviors described using a 4-point Likert scale (never, once, more than once, or many times). The original development of RTSHIA and its psychometric properties were confirmed in a sample of 651 adolescents aged between 12 and 19 years and in a second sample of 71 young people referred to mental health services for self-harm behavior (12–18 years of age). Specifically, the SH factor explains 49.8% of the variance, and the RT factor explains 10.8% of the variance. Together, both factors explain 60.6% of the total variance. To obtain a measure of RT and SH in adolescence adapted to their culture, Xavier and colleagues [36] tested the factorial structure of the Portuguese version of the RTSHIA in a sample composed of 868 community adolescents (12–19 years of age) and confirmed the presence of the same two factors.

Despite the importance of both deliberate self-harm (SH) and risky behavior, such as risk-taking (RT), beyond adolescence, there are currently no validated tools in Italy to assess these behaviors concurrently. Additionally, tools that evaluate RT and SH independently, such as the Deliberate Self Harm Inventory [37,38], are typically validated for adult populations or used without Italian validation, for example, the scale Non-suicidal self-injury proposed by Prinstein and colleagues [39] and Giletta and colleagues [40]. The prevalence of self-harm and risky behavior is increasing and becoming more evident, as seen in Ougrin and colleagues’ investigation [41] of self-harm in adolescents in 10 countries, including Italy. They found that the proportion of teens turning to inpatient emergency departments increased by 50% in 2019 and 57% in 2020, with an increase in those having difficulty with emotion regulation between 58% and 66% likely due to the COVID-19 pandemic in the past two years [30]. These data highlight the importance of investigating these behaviors with culturally appropriate measures [42,43], which are useful in each specific social context, taking into account social and moral norms. Therefore, we tested the Italian validity of the RTSHIA in a cohort of non-clinical Italian adolescents aged between 14 and 21 years.

To achieve this, we first assessed the structure of the Italian version of the RTSHIA (RTSHIA-I; [44]), with an exploratory and a confirmatory factor analysis based on the model proposed by Vrouva and colleagues [32]. We hypothesized that the RTSHIA-I would have the same two-factor structure as both the original English and Portuguese versions. We also examined the reliability and convergent validity by correlating scores from RTSHIA-I with the Difficulties in Emotion Regulation Scale (DERS, [45,46])—evaluating emotion regulation abilities—and the Assessment of Psychopathology in Adolescence Questionnaire (Q-PAD; [47]), assessing psychopathology. Regarding the DERS, we hypothesized a correlation between difficulties in emotion regulation abilities and both RT and SH behaviors. With respect to Q-PAD, we hypothesized a correlation between RT–SH and the indicators of psychopathology specifically referred to externalizing traits for RT (e.g., substance abuse) and internalizing traits for SH (e.g., depression), in line with Vrouva and colleagues’ results [32].

## 2. Methods

### 2.1. Participants

For the study, two groups of students from 9th to 12th Grade were recruited in Lombardia (North of Italy).

The original questionnaire (back-translated into Italian) was administered to the first group of 638 participants (*boys: N = 355, Mean age = 18.17, SD = 1.12, range 14—20.92; girls: N = 283, Mean age = 17.11, SD = 1.55, range 14.08—20.75*) for exploratory analysis. Subsequently, a confirmatory factor analysis (CFA) was conducted on the second group of 660 students (*boys: N = 323, Mean age = 17.10, SD = 1.64, range 14.1—20.42; girls: N = 337, Mean age = 17.24, SD = 1.52, range 14—21.17*). The study had a total of 1298 participants, and the sample size was calculated based on Comrey and Lee’s suggestion [48] that item reduction procedures require a sample size of more than 1000 participants for excellent scale development. Based on a sample of 1298 individuals drawn from the Italian population, a sampling error of approximately 0.014 was calculated, with a confidence level of 95%. The sample standard deviation used to calculate the sampling error was 0.05.

The age range of participants was reflective of the original work by Vrouva et al. [32]. No participant dropped out of the study, and all respondents answered each item. No other exclusion criteria were applied. Participants above 18 years of age and parents or guardians of those under 18 gave written consent to participate in the study without payment.

### 2.2. Measures

#### 2.2.1. Risk Taking and Self-Harm Inventory for Adolescents—Italian Version

The participants were administered the Italian version of the original English RTSHIA scale [44] validated by Vrouva and colleagues [32]. The scale was backtranslated from English to Italian by a professional translator and a psychologist to ensure the meaning of each sentence/item was accurately reflected. The scale consists of a total of 27 items, divided into two dimensions: risk-taking (RT), which includes 8 items related to engaging in dangerous or transgressive behaviors, and self-harm (SH), which includes 19 items related to self-mutilation, self-injury, drug overdose, and suicide attempt.

As described in Vrouva et al.’s [32] original work, the RT-related dimension measures behaviors such as stealing, being expelled from school, staying out late at night without warning, getting into fights, engaging in substance-dependent conduct (alcohol, drugs, tobacco), and having unprotected sex. The SH-related dimension measures behaviors such as cutting, burning, biting, bruising, using acid on the skin, pulling out hair, not eating or overeating, and engaging in self-injurious behaviors that require hospitalization or medical treatment. One item from the RT scale, which investigates the tendency to have sex without precautions, was reinstated based on the recommendation of Vrouva and colleagues as it is considered a major risk behavior among adolescents. Item 27, which measures self-injurious behaviors leading to hospitalization or severe injury, was originally developed by Lundh et al. [49].

The items were ordered gradually in terms of severity and expected frequency (from the mild and frequent to the serious and rare). The original questionnaire and the identified factors have good psychometric properties of consistency, reliability, and validity that highlight how dangerous and self-injurious behaviors are connected, but at the same time, are distinct from each other as they probably refer to different coping processes and aspects of vulnerability.

The items are answered on a 4-point Likert scale, ranging from never to many times, as used by Lundh et al. [49].

The original RTSHIA items in English and Italian are given in the Appendix A.

#### 2.2.2. Difficulties in Emotion Regulation Scale

The *Difficulties in Emotion Regulation Scale* (DERS) [46] is a 36-item, 5-point Likert scale designed to assess difficulties in emotion regulation when negative emotions are experienced. DERS measures the presence of potential difficulties in the awareness and understanding of emotions, the capacity to control impulsive behaviors, and the ability to use flexible emotional regulation strategies appropriate to the context and demands of the situation. The items are assigned to six sub-scales: (a) non-acceptance of emotional responses (Nonacceptance); (b) difficulties engaging in goal-directed behavior (Goals); (c) impulse control difficulties (Impulse); (d) lack of emotional awareness (Awareness); (e) limited access to emotion regulation strategies (Strategies); and (f) lack of emotional clarity (Clarity).

#### 2.2.3. The Assessment of Psychopathology in Adolescence Questionnaire

*The Assessment of Psychopathology in Adolescence Questionnaire (Q-PAD)* [47] is an 81-item, 4-point Likert scale that assesses psychopathology and well-being in juveniles. Q-PAD measures eight different dimensions, including (a) anxiety, (b) depression, (c) body dissatisfaction, (d) substance abuse, (e) interpersonal conflicts, (f) family problems, (g) future uncertainty, (h) psychosocial risk, and finally (i) self-esteem and well-being, thus providing an overall assessment of the individual’s state of adaptation.

### 2.3. Procedure

The survey was conducted in classrooms during daily activities by researchers and included demographic data collection and administration of the RTSHIA-I, DERS, and Q-PAD questionnaires. After collecting demographic data on paper, the three questionnaires were administered in random order among classes. For the RTSHIA-I, participants were informed about the nature of the questions, which covered a variety of actions they may sometimes perform. Participants were reassured that some questions perceived as “odd” served to learn more about specific behaviors that may occur in adolescence. Participants were also reassured that all their answers would be kept confidential, and they should answer truthfully.

The ethics approval was obtained from the ethics committee of the Department of Psychology, Università Cattolica del Sacro Cuore, Milan, Italy, and the procedure met all American Psychological Association ethical principles for the use of human subjects as well as compliance with the guidelines in the Declaration of Helsinki.

### 2.4. Data Analysis

The statistical program SPSS (version 27) and JASP (vs. 0.11.1) [JASP Team (2019)] JASP (Version 0.11.1) [Computer software] were used to analyze the data. Data analysis was carried out to (1) determine the structure of the questionnaire, (2) confirm the structure, and (3) evaluate the convergent validity of the scale.

To examine the factor structure that underpins RTSHIA-I, we conducted both exploratory and confirmatory factor analyses. As described in the Section 2.1 above, two different groups were recruited for the exploratory and confirmatory analyses. The exploratory analysis was carried out via a Principal Component Analyses (PCA), using the data from the 638 respondents. Oblique rotation (direct oblimin) was used because the factors were presumably related to each other rather than independent. Delta was set to 0. In addition, Horn’s method [50] was used to confirm the number to retain. The number of awaited factors was specified before performing the analysis: we expected a two-factor model as in the original work by Vrouva and colleagues [32].

Skewness and kurtosis of the RTSHIA items were first checked to assess normal distribution; West, Finch, and Curran [51] recommend concern if skewness > 2 and kurtosis > 7, and Hair and colleagues [52] defined normal data as having skewness between −2 and +2 and kurtosis between −7 and +7.

To test the reliability of the two-factor structure yielded by the Italian sample, a confirmatory factor analysis (CFA) was conducted on the second group, composed of 660 teenagers. Results were interpreted in terms of goodness of fit of the model, using the following indices: the comparative fit index (CFI), the Root Mean Square Error of Approximation (RMSEA), and the Standardized Root Mean Squared Residual (SRMR). A CFI value greater than or equal to 0.90 is usually considered satisfactory [53,54,55], an RMSEA value ranging from 0.05 to 0.08 reflects an acceptable fit [56], and an SRMR value of 0.08 or less is considered an indicator of good fit [55,57].

The reliability of the models was further examined across sex and grades of school in the second group. Separate CFAs were carried out for boys (N = 323) and girls (N = 337) and middle (Grades 9 and 10, N = 281) and late adolescents (Grades 11, 12, and 13, N = 379). Cronbach’s α coefficient was calculated to examine the internal consistency of the scale, considered globally and in its two dimensions, as yielded by the factor analysis.

Finally, to test convergent validity, the RTSHIA questionnaire was compared with two Italian-validated scales: the DERS questionnaire and the Q-PAD test described above.

## 3. Results

### 3.1. Exploratory Factor Analysis

To investigate the dimensionality of the scale, exploratory factor analysis was initially carried out using Principal Component Analysis, and the final factorial solution was obtained through a direct oblimin rotation. Sample adequacy and appropriateness of the PCA were confirmed by the value of the Kaiser–Meyer–Olkin statistic (KMO 0.971) and results of Bartlett’s test of sphericity (*p* < 0.001). The exploratory factor analysis was carried out using all 27 items; no items were removed. A two-factor solution was revealed, with eigenvalues over 1, accounting for 66.6% of the variance. As in Vrouva and colleagues’ work (2010), the two factors are labeled as Self-Harm behaviors (SH) and Risk-Taking behaviors (RT).

The interfactor correlation was 0.55. The component matrix showed a dominant first factor (explaining 57.9% of the variance), with 19 out of 27 items having rotated loadings reaching 0.53 or higher, and a second factor (accounting for 9.03% of the variance) determined by the remaining 8 items having loadings of 0.45 or higher. Table 1 displays the main dimensions that emerged from the PCA.

**Figure 1:**
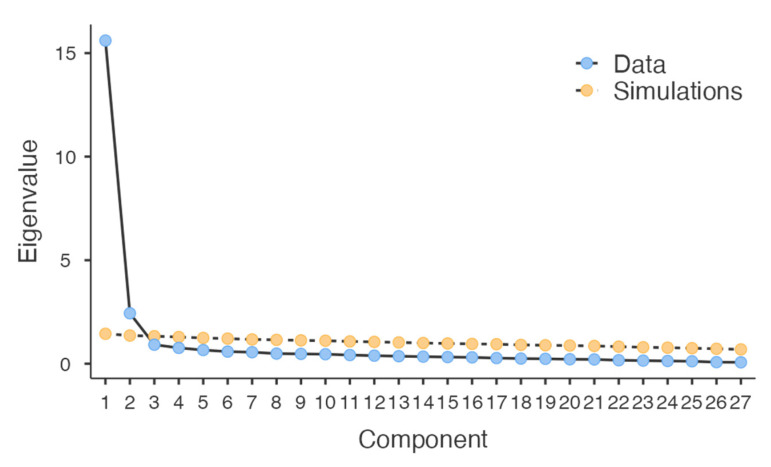
Scree plot resulting from the parallel analysis.

To confirm this, Horn’s parallel analysis suggested that the same two factors should be retained, explaining 51.3% and 15.5% of the variance for SH and RT, respectively, and cumulatively explaining 66.8% of the total variance. Thus, the questionnaire structure obtained from the PCA was confirmed by the results of the parallel analysis (Figure 1).

### 3.2. Descriptive Analysis of RTSHIA Items

The descriptive analysis of the RTSHIA items is presented in Table 2. The mean of the responses to the 27 items ranged from 0.38 to 1.31 (*SD* MIN = 0.52; *SD* MAX = 1.15). Furthermore, in line with the recommendations of West, Finch, and Curran (1990) and Hair et al. (2010), the results showed that most items had a normal distribution (skewness MIN = 0.65, skewness MAX = 2.2; kurtosis MIN = −0.31, kurtosis MAX = 7.69).

### 3.3. Confirmatory Factor Analysis

**Figure 2:**
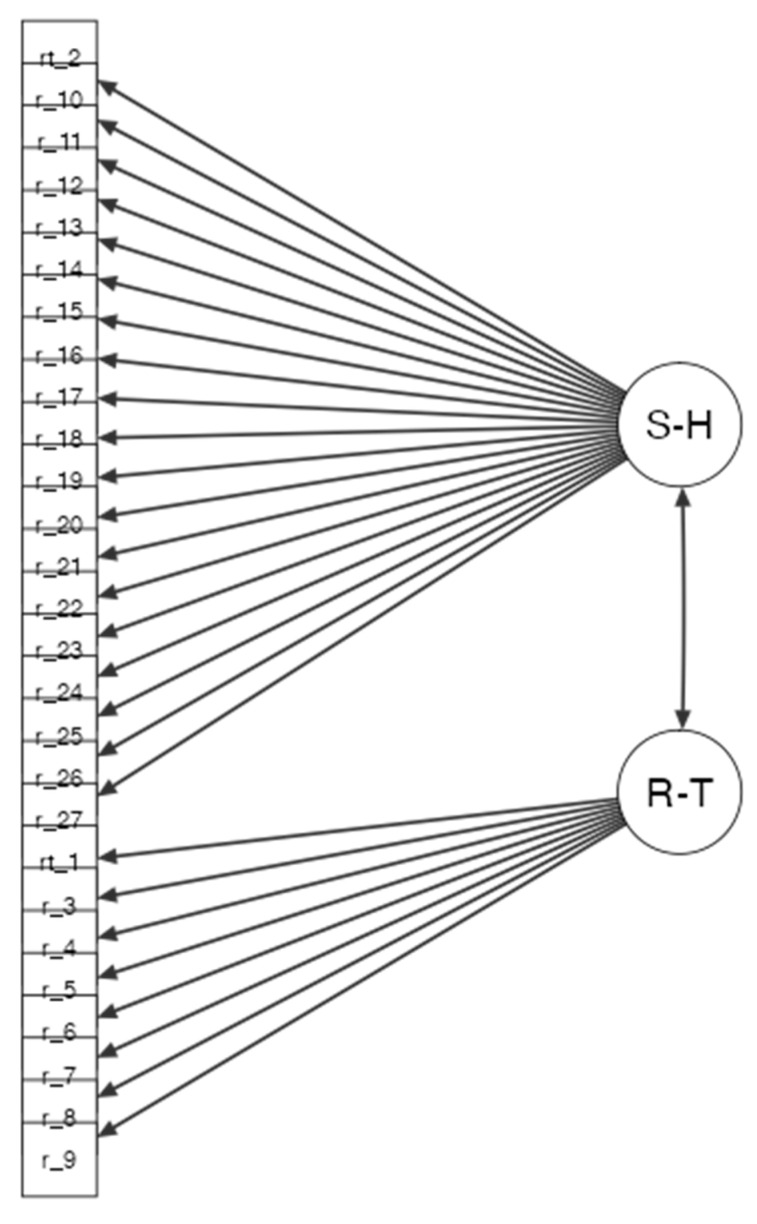
Graphical summary of the Confirmatory Factor Analysis obtained from the 27 items of the Risk Taking and Self Harm Inventory for Adolescents (RTSHA-I) (N = 654).

A Confirmatory Factor Analysis (CFA) was carried out to assess whether the theoretical model fitted the data. The two-factor structure of RTSHIA-I was tested, and we carried out the CFA with the other sample group (N = 660). Table 3 presents the fit indices. A careful inspection of modification indices indicated that the model could be improved if some items were correlated. The new CFA (see Figure 2) showed better fit indices, with both The Comparative Fit Index (CFI) and the Standardized Root Mean Squared Residual (SRMR) considered acceptable because they were, respectively, larger than 0.90 and greater than 0.50. The root-mean-square error of approximation (RMSEA) nearly met the recommended cut-off value of 0.08 (see Table 4).

In order to confirm the reliability of the model, two further CFAs were carried across sex and grades of school (middle: Grades 9 and 10, N = 281; late adolescents: Grades 11, 12 and 13, N = 379). As shown in Table 5 and Table 6, most of the indices was close to the recommended cut-off values.

### 3.4. Reliability and Validity Analyses

The scale reliability and validity were examined. First, the reliability of the scale and its subscales was tested. Results showed satisfactory internal consistency. The global scale α coefficient was 0.96. The alpha for the SH scale and RT scale was also high at 0.96 and 0.86, respectively.

In the next set of analyses, we assessed the convergent validity of the RTSHIA-I by examining its correlation (Pearson r) with theoretically related measures, namely the DERS and the Q-PAD questionnaires. The correlations between the RT and SH subscales and the relevant measures are presented in Table 7.

As shown in Table 6, the RT and SH subscales generally correlated with both DERS and Q-PAD scales, except for Q-PAD future uncertainty and DERS clarity subscales, which did not correlate with either of the RTSHA-I subscales. Both RT and SH correlated positively with the Q-PAD Family Problems and Psychosocial Risk scales and with the DERS Awareness subscale.

Regarding RT, the highest correlation was found between RT and the Q-PAD Substance Abuse scale (medium effect size, r = 0.51; Cohen, 1992), which, in contrast, failed to correlate significantly with SH. A similar correlation was found in Vrouva and colleagues’ original work (2010), in which the Risk-Taking subscale correlated highly and positively with the MACI Substance Abuse scale. Additionally, RT correlated negatively with the DERS Non-Acceptance subscales.

SH correlated positively with the Q-PAD Depression, Interpersonal Conflicts, and Body Dissatisfaction scales, as well as with the DERS Impulse subscale, and negatively with Q-PAD Self-esteem and Well-being scales. In contrast to RT, SH also correlated positively with Q-PAD Anxiety and DERS Impulse.

## 4. Discussion

The aim of this study was to examine the psychometric properties of the RTSHIA-I in adolescents. Specifically, we first tested the factorial structure of the scale, then its reliability and convergent validity by examining its associations with RTSHIA-I, emotion regulation difficulties, and psychopathological indexes.

With respect to the structure analysis, our data replicate the factorial structure of the original [32] and Portuguese [36] versions of the scale, confirming the presence of two factors: Risk-Taking and Self-Harm. As in the original scale, the Risk-Taking factor includes items related to putting oneself in dangerous situations, staying out late at night, aggressive behavior, sexual promiscuity, and use of alcohol/drugs/smoking. The Self-Harm scale encompasses the presence of intentional behavior such as cutting, burning, biting, beating, scratching, tearing one’s hair out, suicidal behavior, hurting oneself emotionally/affectionally, and refusing food.

As outlined above, the bifactorial structure was demonstrated via CFA, with indices reaching the recommended cutoff values. Additionally, to investigate the efficacy of the model across sex and age, separated CFAs were carried out for girls and boys, and for the biennium and triennium classes. Although the goodness-of-fit indices were not always strictly within the acceptable range, when considered together, they were adequately close to supporting the two-factor model.

Although the original and the Italian versions of the RTSHIA globally overlap, we have identified two main differences between them. First, item 2, “Have you ever been suspended (punished by being banned from class) or expelled from school?” was originally part of the Risk-Taking factor in the English version, but in RTSHIA-I, it has been moved to the Self-harm factor. In the Italian context, suspension from school is an exceptional decision, that is taken after a series of negative and transgressive behaviors, and after warning the student several times. Therefore, this measure can be perceived as linked to self-harm behavior because the student is usually aware of the consequences of their behavior, which they most likely activate intentionally with the aim of being expelled from the educational system. The Portuguese version of the RTSHIA has removed this question, possibly due to cultural differences.

The second difference between the original and RTSHIA-I relates to item 6: “Have you ever had sex avoiding precautions against sexually transmitted diseases or pregnancy?”. This item has been removed in the original version because it equally loaded on both RT and SH factors, whereas in the Italian version, it clearly loads on the Risk-Taking factor, indicating that Italian adolescents specifically perceived it as a risk. Educators in Italy frequently use the word “risk” when discussing such sexual behaviors with adolescents, for example, in school programs aimed at preventing sexually transmitted diseases. Moreover, sex is a pleasurable behavior that can have unintended negative consequences, unlike self-injurious behaviors that cause physical pain in the first place. The presence of this item in the Risk-Taking factor of the Portuguese version suggests possible cultural differences that might characterize Anglo-Saxon versus Mediterranean countries. Further investigation into these cultural differences would be interesting.

Correlations between RTSHIA-I and questionnaires investigating emotion regulation difficulties and psychopathological indexes confirm the validity of the RTSHIA-I. Both identified factors, Risk-Taking and Self-Harm, show positive correlations with a lack of emotional awareness, which is in turn correlated with emotional regulation abilities, and with family problems, psychosocial risk, and interpersonal conflicts, which are related to the assessment of psychopathology. The first correlation highlights the important role of emotional awareness in Risk-taking and Self-harm. Both are characterized by limited emotional awareness: when the adolescents are guided by emotion-driven impulsiveness [9], they can be engaged in risk-taking behaviors; when the adolescents do not recognize their own emotional states, they can try to calm an undetectable emotional pain by causing physical harm.

In assessing psychopathology, the family problems component outlines the adolescent’s discomfort in the family, feelings of not being understood, and difficulty relating to parents. The correlation between the RTSHIA-I and this component is in line with the findings about the important role of the family in self-harm behavior; Aggarwal and colleagues [58], for example, found that family conflict is a risk factor for self-harm, while they identify family understanding as a protective factor. Moreover, the RTSHIA-I is correlated with the psychosocial risk component, indicating discomfort in interpersonal relationships, risky behavior (e.g., alcohol abuse), and uncertainty about the future, and with the interpersonal conflict component, indicating the presence of dysfunctional relationships and feelings of inadequacy. These results also indicate the role of mentalization difficulties in risk-taking and self-harm behaviors. The family and social components play an important role in the development of mentalization. Mentalization originates from attachment relationships with familiar caregivers [59], which are built on early emotional interactions between the infant and the adult. A failure of emotional mirroring in the dyad may be followed by difficulty in mentalizing correctly, which may lead to maladaptive behaviors, such as self-harm [27,60]. Evidence suggests that attachment style affects the development of mentalization in both familiar [61] and extrafamilial settings (e.g., at school) [62,63]. The discomfort and conflicts expressed by adolescents in family and extrafamilial contexts suggest dysfunctional attachment relationships that fail to support emotional regulation and the development of mentalization abilities in the child, thus fostering the emergence of risky and self-harm behaviors later in life.

The correlational results partially support the hypothesis linking risk-taking with externalizing behaviors and self-harm with internalizing behaviors. Specifically, adolescents who engage in risk-taking tend to abuse substances [32,64] and have difficulty accepting and managing their emotional reactions, particularly distress. It appears that their difficulty in focusing on negative emotions leads them to seek external modes of emotional regulation, which then results in the activation of risky behaviors. The correlations related to self-harm are more complex. The results suggest that adolescents who engage in self-harm tend to exhibit high levels of internalizing problems, such as anxiety, depression, and body dissatisfaction. They also show low levels of self-esteem and well-being, poor knowledge of emotion regulation strategies, and difficulty in adopting goal-oriented behaviors. At the same time, they have difficulties with impulse control and show high levels of interpersonal conflict, indicating externalizing problems. Cerutti and colleagues [21] showed, for the first time, that self-harm in the nonclinical population is not only related to internalizing behaviors but also to externalizing behaviors. More recently, Martínez-Ferrer and Stattin [65] assumed that a mix of mutually hostile relationships with others in different everyday contexts and psychosocial difficulties (i.e., anxiety and depression) may underlie the activation of this type of behavior. Similarly, Latina and Stattin [66] suggest that the experience of stressful interpersonal relationships can activate both internalizing and externalizing reactions. Based on these premises, future research could investigate the role of attachment with extrafamilial caregivers or peers/friends in self-harm behaviors, focusing particularly on the stress generated by insecure attachment bonds.

### 4.1. Limitations

The first limit of this study concerns the sample. A convenience sample was recruited for this work by contacting schools willing to collaborate, and this prevented us from having a-priori information on the sampling frame. Moreover, this sample was recruited in the north of Italy and is not representative of the Italian population. Although data on regional differences in the prevalence of the phenomena analyzed are not available to date, given the high number of risk factors, such as socio-economic disadvantage and psychiatry illnesses, it cannot be ruled out that RT and SH have different prevalence in different parts of Italy. Therefore, in the future, it will be interesting to apply RTSHIA-I to central and southern Italy as well.

The lack of an additional sample with independently identified clinical criteria did not allow us to select an appropriate cut-off point for the two scales of the RTSHIA-I. Another critical aspect of the research is the cross-sectional nature of the validation, which did not allow for a longitudinal assessment of the subjects’ development over time. Although the structure of the RTSHIA-I appears to be confirmed in both early and late adolescence, a future longitudinal study with a larger sample might confirm the structure with a greater level of certainty. Furthermore, as in the original work, the discriminant validity was not carried out. However, self-esteem and well-being of Q-PAD and non-acceptance of DERS, which are discriminant items, are negatively correlated with the RTSHIA factors. This result is encouraging regarding the ability of the RTSHIA to correctly measure the constructs of risk taking and self-harm.

Finally, as pointed out by Vrouva and colleagues [32], the RTSHIA is a self-report measure, and therefore, the quality of answers depends on the willingness to respond to such sensitive questions. Although the adolescents involved in this research seemed curious and motivated, and the researchers answered all questions and clarified any doubts, we cannot rule out the possibility that some participants did not answer completely truthfully to avoid sharing personal information. Nonetheless, we believe that using the RTSHIA-I in individual interactions with a researcher or a clinician may facilitate the expression of adolescents compared to the collective procedure applied in this research.

### 4.2. Conclusions

The present study validates the Italian version of the RTSHIA, which assesses both Risk-taking and Self-harm behaviors in adolescence. The RTSHIA-I has several advantages over questionnaires that evaluate these behaviors separately, both in research and clinical field. Firstly, information on interrelated behaviors can be obtained on a single scale. Additionally, assessing both risk-taking and self-harm behaviors using a single measure is convenient, economical, and easy to propose to youth populations without overt psychopathology, as highlighted by Uh and colleagues [22] and Xavier and colleagues [36]. Another strength of the scale is its response mode, which not only highlights the presence of RT and/or SH behaviors, but also their likelihood of occurrence, contributing to an accurate profile of the subject.

As suggested by Muehlenkamp and colleagues [17], administering a comprehensive assessment to detect these behaviors can help screen adolescents with psychopathology, distinguishing them from those who have experienced RT and SH as part of typical development. This can be the starting point for a clinical intervention that simultaneously considers both types of behaviors, providing the opportunity to act promptly and comprehensively for the adolescent’s well-being. In this regard, the availability of the Italian version of the RTSHIA will facilitate more accurate assessments of the treatment effects of cultural-tailored interventions or prevention strategies (for the relevance of the cultural differences see, for example, [67]). The assessment of risk-taking and self-harm behavior is relevant also from a preventive perspective: studying the impact on the severe restrictions due to COVID-19 in Italy, De Luca and colleagues [68] found that the adolescents considered vulnerable in the pre-pandemic period are at high risk of engaging in SH in the post-pandemic period. This suggests that having this tool validated for a specific culture allows early detection of vulnerable adolescents, facilitating a supportive intervention aimed to better manage possible stressful situations in the future.

## Figures and Tables

**Table 1:** Component Loadings for 27 items RTSHIA-I.

RTSHIA Item	Factor 1 SELF-HARM	Factor 2 RISK-TAKING
Item 1		0.658
Item 2	0.531	
Item 3		0.785
Item 4		0.447
Item 5		0.525
Item 6		0.534
Item 7		0.843
Item 8		0.827
Item 9		0.732
Item 10	0.920	
Item 11	0.847	
Item 12	0.876	
Item 13	0.834	
Item 14	0.903	
Item 15	0.884	
Item 16	0.930	
Item 17	0.856	
Item 18	0.715	
Item 19	0.821	
Item 20	0.863	
Item 21	0.592	
Item 22	0.872	
Item 23	0.839	
Item 24	0.870	
Item 25	0.856	
Item 26	0.916	
Item 27	0.897	

Note. ‘Principal component analysis’ extraction method was used in combination with a direct oblimin rotation.

**Table 2:** Mean, standard deviation, skewness, and kurtosis of the RTSHIA items.

	Mean	Standard deviation	Skewness	Kurtosis
Item 1	1.30	1.097	0.650	−0.310
Item 2	0.45	0.620	1.551	3.939
Item 3	0.99	0.935	0.890	0.389
Item 4	0.67	0.894	1.575	2.631
Item 5	0.52	0.746	2.202	7.686
Item 6	0.67	0.854	1.931	5.640
Item 7	0.90	0.916	1.042	0.907
Item 8	0.77	0.926	1.439	2.202
Item 9	1.31	1.150	0.776	−0.133
Item 10	0.49	0.738	1.940	5.216
Item 11	0.41	0.586	1.362	2.219
Item 12	0.47	0.670	1.758	4.500
Item 13	0.49	0.698	1.478	2.313
Item 14	0.53	0.766	1.978	5.718
Item 15	0.49	0.693	1.687	3.707
Item 16	0.40	0.586	1.882	7.350
Item 17	0.47	0.710	2.129	7.278
Item 18	0.47	0.684	1.718	4.064
Item 19	0.63	0.838	1.639	3.214
Item 20	0.40	0.550	1.099	1.153
Item 21	0.70	0.886	1.431	2.152
Item 22	0.60	0.838	1.926	4.905
Item 23	0.39	0.522	0.857	−0.076
Item 24	0.58	0.834	1.780	3.579
Item 25	0.59	0.855	1.839	4.054
Item 26	0.39	0.553	1.344	3.025
Item 27	0.38	0.530	1.200	1.882

**Table 3:** Fit Measures without Modification Indices.

			RMSEA 90% CI	
**CFI**	**SRMR**	**RMSEA**	**Lower**	**Upper**
0.898	0.053	0.096	0.092	0.099

**Table 4:** Fit Measures with Modification Indices.

			RMSEA 90% CI	
**CFI**	**SRMR**	**RMSEA**	**Lower**	**Upper**
0.930	0.050	0.081	0.077	0.085

**Table 5:** Fit indices—CFA across sex.

Index	Value
Comparative Fit Index (CFI)	0.911
Root mean square error of approximation (RMSEA)	0.094
Standardized root mean square residual (SRMR)	0.050

**Table 6:** Fit indices—CFA across grades of school.

Index	Value
Comparative Fit Index (CFI)	0.891
Root mean square error of approximation (RMSEA)	0.073
Standardized root mean square residual (SRMR)	0.076

**Table 7:** Correlations with Q-PAD and DERS.

	Self-harm Pearson Correlation	Risk-taking Pearson Correlation
Q-PAD body dissatisfaction	0.220 **	−0.040
Q-PAD anxiety	0.334 **	0.093
Q-PAD depression	0.226 **	0.110
Q-PAD substance abuse	0.059	0.511 **
Q-PAD interpersonal conflicts	0.329 **	0.157 *
Q-PAD family problems	0.291 **	0.217 **
Q-PAD future uncertainty	0.110	−0.023
Q-PAD psychosocial risk	0.286 **	0.440 **
Q-PAD self-esteem and well-being	−0.283 **	−0.304
DERS non-acceptance	−0.069	−0.144 **
DERS goals	0.151 **	0.084
DERS impulse	0.254 **	0.068
DERS awareness	0.284 **	0.260 **
DERS strategies	0.161 **	0.039
DERS clarity	0.079	−0.047

** Correlation is significant at the 0.01 level (2-tailed). * Correlation is significant at the 0.05 level (2-tailed).

## Data Availability

The raw data supporting the conclusions of this article are available from the authors on request.

## Appendix A. Risk-Taking and Self-Harm Inventory for Adolescents

### Appendix A.1. Instructions

This questionnaire asks about a number of different things that young people sometimes do. Please do not be concerned if some statements seem unusual. They are included to provide us with greater understanding and knowledge about these behaviors and the best way to help young people.

- Please complete this questionnaire on your own.
- If a statement is not applicable to you, please circle Never.
- You do not have to answer any questions that you prefer not to answer.
- Please try to answer as truthfully as possible.
- All your answers are kept strictly confidential.

1	*Have you ever put yourself in a risky situation (such as classroom cheating, traveling without a valid ticket, shoplifting, etc.) knowing that you may get caught?*	Never	Once	More than once	Many times
1	Ti sei mai messo in una situazione pericolosa (ad esempio, saltare la scuola, viaggiare senza biglietto, rubare in un negozio), sapendo di poter essere scoperto?	Mai	Una volta	Più di una volta	Molto spesso
2	*Have you ever been suspended (i.e., punished with exclusion) or dropped out of school?*	Never	Once	More than once	Many times
2	Sei mai stato sospeso (punito con il divieto di entrare in classe) o espulso da scuola?	Mai	Una volta	Più di una volta	Molto spesso
3	*Have you ever stayed out late at night, without your parents knowing where you are?*	Never	Once	More than once	Many times
3	Sei mai stato fuori fino a tarda notte, senza che i tuoi genitori sapessero dov’eri?	Mai	Una volta	Più di una volta	Molto spesso
4	*Have you ever participated in gang violence or physical fights or held a weapon?*	Never	Once	More than once	Many times
4	Hai mai partecipato a una rissa, fatto a botte o tenuto in mano un’arma?	Mai	Una volta	Più di una volta	Molto spesso
5	*Have you ever been promiscuous (i.e., had many sexual partners within a short period of time)?*	Never	Once	More than once	Many times
5	Ti è mai capitato di fare sesso con più persone nel giro di poco tempo?	Mai	Una volta	Più di una volta	Molto spesso
	-				
6	Hai mai fatto sesso senza proteggerti dal rischio di prendere qualche malattia o di una gravidanza?	Mai	Una volta	Più di una volta	Molto spesso
7	*Have you ever had so much alcohol that you were really drunk?*	Never	Once	More than once	Many times
7	Hai mai bevuto alcolici fino ad essere completamente ubriaco?	Mai	Una volta	Più di una volta	Molto spesso
8	*Have you ever used drugs (such as marijuana, cocaine, LSD, etc.)?*	Never	Once	More than once	Many times
8	Hai mai fatto uso di droghe (come marijuana, cocaina, LSD o altro)?	Mai	Una volta	Più di una volta	Molto spesso
9	*Have you ever smoked tobacco?*	Never	Once	More than once	Many times
9	Hai mai fumato tabacco?	Mai	Una volta	Più di una volta	Molto spesso

Please say yes to the following questions only if you did the behavior intentionally, or on purpose, to hurt yourself. Circle *Never* if you did something only accidentally (e.g., you tripped and banged your head on accident).

10	*Have you ever intentionally cut your skin?*	Never	Once	More than once	Many times
10	Ti sei mai procurato intenzionalmente dei tagli?	Mai	Una volta	Più di una volta	Molto spesso
11	*Have you ever intentionally burned yourself with a hot object (such as a cigarette)?*	Never	Once	More than once	Many times
11	Ti sei mai fatto volontariamente una bruciatura con qualcosa di rovente (come per esempio una sigaretta accesa)?	Mai	Una volta	Più di una volta	Molto spesso
12	*Have you ever intentionally bitten yourself, to the extent that you broke the skin?*	Never	Once	More than once	Many times
12	Ti sei mai morsicato da solo, fino al punto di lacerare la pelle?	Mai	Una volta	Più di una volta	Molto spesso
13	*Have you ever intentionally banged your head against something or hit or punched yourself, to the extent that you caused a bruise to appear?*	Never	Once	More than once	Many times
13	Ti sei mai fatto venire un livido o un bernoccolo sbattendo intenzionalmente la testa contro qualcosa oppure colpendoti da solo?	Mai	Una volta	Più di una volta	Molto spesso
14	*Have you ever intentionally prevented wounds from healing or picked at areas of your body to the point of drawing blood?*	Never	Once	More than once	Many times
14	Hai mai fatto qualcosa per evitare che una ferita guarisse, o ti sei mai ferito da solo in una qualsiasi parte del corpo?	Mai	Una volta	Più di una volta	Molto spesso
15	*Have you ever intentionally scraped, scrubbed, or scratched your skin to the point of breaking your skin or drawing blood?*	Never	Once	More than once	Many times
15	Hai mai raschiato, rimosso o graffiato volontariamente la tua pelle fino al punto di farla sanguinare?	Mai	Una volta	Più di una volta	Molto spesso
16	*Have you ever intentionally rubbed a sharp object (such as sandpaper) or dripped anything toxic (such as acid) onto your skin?*	Never	Once	More than once	Many times
16	Hai mai strofinato volontariamente un oggetto tagliente (per esempio, carta vetrata) o versato qualcosa di tossico (come un acido) sulla tua pelle?	Mai	Una volta	Più di una volta	Molto spesso
17	*Have you ever exercised an injured part of your body intending to hurt yourself?*	Never	Once	More than once	Many times
17	Hai mai forzato una parte del tuo corpo che ti faceva male, con lo scopo di fartene ancora?	Mai	Una volta	Più di una volta	Molto spesso
18	*Have you ever intentionally pulled your hair out?*	Never	Once	More than once	Many times
18	Ti sei mai strappato i capelli di proposito?	Mai	Una volta	Più di una volta	Molto spesso
19	*Have you ever starved yourself to hurt or punish yourself?*	Never	Once	More than once	Many times
19	Hai mai rifiutato di mangiare allo scopo di punire te stesso?	Mai	Una volta	Più di una volta	Molto spesso
20	*Have you ever forced yourself to eat too much to hurt or punish yourself?*	Never	Once	More than once	Many times
20	Ti sei mai obbligato a mangiare così tanto da star male, con lo scopo di provocarti dolore o di punire te stesso?	Mai	Una volta	Più di una volta	Molto spesso
21	*Have you ever stayed in a friendship or a relationship with somebody who repeatedly hurt your feelings on purpose?*	Never	Once	More than once	Many times
21	Sei mai stato coinvolto in un’amicizia o in una relazione con qualcuno che feriva ripetutamente e di proposito i tuoi sentimenti?	Mai	Una volta	Più di una volta	Molto spesso
22	*Have you ever tried to make yourself suffer by thinking horrible things about yourself?*	Never	Once	More than once	Many times
22	Hai mai tentato di provocarti dolore pensando cose orribili di te stesso?	Mai	Una volta	Più di una volta	Molto spesso
23	*Have you ever taken an overdose? (i.e., taken an excessive amount of medication without having been prescribed this dosage)*	Never	Once	More than once	Many times
23	Hai mai preso un’overdose (per esempio, un quantitativo eccessivo di un farmaco, senza che quel dosaggio ti fosse prescritto)?	Mai	Una volta	Più di una volta	Molto spesso
24	*Have you ever seriously thought about harming a part of your body?*	Never	Once	More than once	Many times
24	Hai mai pensato sul serio di farti fisicamente del male?	Mai	Una volta	Più di una volta	Molto spesso
25	*Have you ever seriously thought about killing yourself?*	Never	Once	More than once	Many times
25	Hai mai pensato di toglierti la vita?	Mai	Una volta	Più di una volta	Molto spesso
26	*Have you ever tried to kill yourself?*	Never	Once	More than once	Many times
26	Hai mai tentato di ucciderti?	Mai	Una volta	Più di una volta	Molto spesso
27	*Have you ever intentionally hurt yourself in any of the abovementioned ways so that it led to hospitalization or injury severe enough to require medical treatment?*	Never	Once	More than once	Many times
27	Ti sei mai provocato delle lesioni in uno dei modi descritti sopra, fino al punto di dover andare in ospedale o di dover ricorrere a cure mediche?	Mai	Una volta	Più di una volta	Molto spesso
